# Heterochromatin protein 1 (HP1) of *Schistosoma mansoni*: non-canonical chromatin landscape and oviposition effects

**DOI:** 10.1590/0074-02760240075

**Published:** 2025-03-31

**Authors:** Natália Silva da Trindade, Marilia Bergamini Valentini, Anne Rognon, Tiago Manuel Fernandes Mendes, Matheus de Souza Gomes, Silmara Marques Allegretti, Christoph Grunau, Fernanda Janku Cabral

**Affiliations:** 1 Universidade Estadual de Campinas, Instituto de Biologia, Departamento de Biologia Animal, Campinas, SP, Brasil; 2 Hosts-Pathogens-Environments Interactions, University of Perpignan Via Domitia, Centre National de la Recherche Scientifique, Institut français de Recherche pour l’Exploitation de la Mer, University of Montpellier, Perpignan, France; 3 Universidade Federal de Uberlândia, Patos de Minas, MG, Brasil

**Keywords:** heterochromatin protein 1, HP1, ChIPmentation, cercariae, sporocysts, Schistosoma mansoni

## Abstract

**BACKGROUND:**

Heterochromatin protein 1 (HP1) is widespread in several organisms playing a role in control of gene expression by heterochromatin formation and maintenance of silent chromatin. *Schistosoma mansoni* is a human parasite that is responsible for Schistosomiasis, a tropical neglected disease in the tropical and subtropical areas in the world, where the intermediate host *Biomphalaria glabrata* is present.

**OBJECTIVES:**

In this study we attempted to investigate if the SmHP1 is enriched in *S. mansoni* chromatin in cercariae larvae stage, compared with another larvae stage sporocysts and its importance for *S. mansoni* life cycle progression and parasite oviposition.

**METHODS:**

We used ChIPmentation with commercial antibody ab109028 that passed in-house quality control. We also used RNA interference, mice infection and histology.

**FINDINGS:**

Our data show that *S. mansoni* HP1 enrichment is non-canonical with a peak at the transcription end sites of protein coding genes. We did not find strong differences in SmHP1 chromatin landscapes between sporocysts and cercariae. Knock- down of SmHP1 in schistosomula and *in vivo* experiments in mice unexpectedly increased parasite oviposition.

**MAIN CONCLUSIONS:**

Our results suggest that SmHP1 may influence chromatin structure in a non-canonical way in *S. mansoni* stages and may play a role in regulation of parasite oviposition.

Schistosomes are trematode parasites responsible for causing schistosomiasis. It is estimated that in 2021, approximately 251.4 million people required treatment.^1^ Intestinal schistosomiasis is caused by *Schistosoma mansoni*. The parasite has a complex life cycle that includes two hosts, snails of the genus *Biomphalaria* and humans, which are intermediate and definitive hosts, respectively.^2^ During its life cycle, the parasite goes through significant stage changes, and it is known that histone post-translational modifications play important roles at each stage of the life cycle. The molecular complexity of *S. mansoni* life cycle has been revealed over many years and efforts to elucidate the *S. mansoni* epigenome has given some insights about epigenetic regulation of the life cycle.^3^ ^,^ ^4^ ^,^ ^5^ ^,^ ^6^ However, it is expected that coregulators will also be required for the maintenance of each stage. In several organisms, the heterochromatin protein 1 (HP1) acts as co-regulators and performs fundamental functions such as maintaining the silenced state of chromatin, DNA repair, among other diverse functions.

This protein is composed of the chromodomain (CD) and chromoshadow (CDS) domains and a linker region between them. CD recognises and binds to methylated histone tails, while CDS is responsible for homo - and heterodimerisation.^7^ The region between the domains interacts with nucleic acids and shows considerable variation across organisms. In contrast, the domains themselves have well-conserved amino acid sequences across different species. In many organisms HP1 is essential for heterochromatin production and gene silencing. This function was described in model organisms such as cancer progression in *Homo sapiens*, *Drosophila melanogaster*, *Plasmodium falciparum*, fission yeast and *Arabidopsis thaliana*.^8^ ^,^ ^9^ ^,^ ^10^ ^,^ ^11^ ^,^ ^12^ Histone modifications are associated with different chromatin states and play important roles in regulating gene expression. *E.g.* in *P. falciparum* methylation of histone H3 at lysine 9 (H3K9) forms binding sites for the HP1 protein and is an important means of controlling gene expression.^10^ ^,^ ^13^ In *Drosophila*, there are three isoforms of HP1. While initially identified as an important part of the heterochromatin through H3K9me3 binding,^14^ it was later found to be also associated with active gene expression.^15^ Metagene profiles in^15^ indicated enrichment of all three HP1 around the transcription start of genes in *Drosophila*.

For *S. mansoni* we showed in a previous study^16^ that HP1 is co-immunoprecipitated with other important DNA associated proteins such as helicases, transcription factors and methyltransferases. These results suggest an important role for HP1 in regulating gene transcription in *S. mansoni*. Geyer and colleagues also described, through *in vitro* experiments with adult worms that SmCBX (Smp_179650, SmHP1, Sm Chromobox protein homolog 5) plays a role in the parasite biology regulating oviposition.^17^ We hypothesised that the HP1 homolog of *S. mansoni* is associated with DNA and plays a role in chromatin structure biology in the parasite life cycle. To test this hypothesis about the involvement of HP1 in the chromatin formation and maintenance in larvae stages, cercaria and sporocyst, were performed the ChIPmentation using an antibody against HP1 homolog used previously.^16^ In addition, for investigation of the role of HP1 in parasite development, migration, fitness, and inflammatory response, we generated *Sm*HP1 *in vivo* Knock-down parasites. Our results suggested that *Sm*HP1 may have a function to regulate epigenetic plasticity in the parasite through the increasing parasite oviposition without affecting host inflammatory response.

## MATERIALS AND METHODS

*Extraction of sporocysts and cercariae* - Sporocysts were obtained from *Biomphalaria glabrata* strain BgGUA, six months infected with *S. mansoni* strain SmDFO. Dissection was performed at room temperature. The snails were placed individually in large petri dishes and water was added. With the help of smaller glass plates, the snails were crushed, and fragments and tissues were removed. The sporocysts were transferred to new petri dishes and the membrane surrounding them was removed. Then, the hepatic pancreas was removed, and two samples were obtained for each snail. While care was taken to dissect sporocysts that were free of host tissue, contaminations with *Biomphalaria* tissues could not be avoided completely. Sporocyst samples were stored at -20ºC.

Cercariae strain SmVEN was obtained from snails *B. glabrata*. The snails were placed in pots of water and exposed to artificial light for 2 h. Next, the cercariae were counted and divided into aliquots with 1000 cercariae each. They were then stored at -20ºC.

Experiments were done according to the national ethical standards. The IHPE animal facility holds agreement number F6613601 and has authorisation APAFIS #39910- 2022121915564694 v2 for the routine production and shared use of *S. mansoni* larvae.

*ChIPmentation S. mansoni cercariae, sporocysts, B. glabrata and Drosophila* - ChIPmentation Kit for Histones (Diagenode, Cat. No. C01011009) was used. Approximately 1000 cercariae, 1 sporocyst and a corresponding amount of *Drosophila* embryos were used for each ChIPmentation reaction. Samples were thawed, resuspended in 500 μL of HBSS, crushed for 1 min with a sterile pestle, and then left at room temperature for 3 min. After this, 13.5 μL of 37% formaldehyde was added and gently homogenised for 10 min at room temperature. To stop the cross-link reaction, 57 μL of glycine from the Diagenode kit was added and left under agitation for 5 min. We then proceeded according to the supplier’s manual. Chromatin was disrupted by sonication using the Bioruptor Pico with five cycles of 30 s ON and 30 s OFF. ChIPmentation was done on an IP-Star pipetting robot according to the pre-established protocol with the modification of washing time to 20 min. Antibody titration was done as described in16 with 0, 2, 4, 8 and 16 µL antibody to obtain the saturating quantity and finally 8 µL of antibody HP1 AbCam (ab109028, lot GR38873387336-9) were used for each reaction. Input libraries were generated as described earlier and the optimal number of library amplification cycles was determined by quantitative polymerase chain reaction (qPCR) as described in the same protocol.^18^

Primers and low molecular weight fragments were removed from the libraries with AMPure beads on the IP-Star and the quality and quantity of the sequencing libraries were checked with a BioAnalyzer High Sensitivity DNA Assay. Sequencing was done by the BioEnvironnement core facility on an Illumina NextSeq 550 as 75 bp single end reads.

*Bioinformatic analyses* - Analyses were carried out on a local Galaxy instance. First, the quality of the sequences were checked by FastQC/MultiQC, adapter sequences were removed with Trim Galore! and reads were aligned to version 9 of the *S. mansoni* genome (schistosoma_mansoni.PRJEA36577.WBPS17.genomic.fa) with permission from parasite wormbase^19^ using Bowtie2 evoking sensitive end-to-end. Uniquely aligned reads were retained using the Bowtie tag”: “XS:”. PCR duplicates were removed with SamTools RmDup. The number of aligned unique reads was down sampled to 4.7 Mio reads per library using Picard Tools [Supplementary data (Table I)]. Differential analysis was done with ChromstaR^20^ with bin size 1000 bp and step size 500 bp.

Annotations came from schistosoma_mansoni.PRJEA36577.WBPS17.genes.gff3. For metagene analysis, the gene feature was retained and 4898 genes of the forward strand were used.

*Differential chromatin states were detected with ChromstaR and default parameters* - Peakcalling was performed also with MACS2 and default parameters, followed by MACS2 bdgbroadcall, with 2 and 5 as -cutoff value. The score in a bedGraph file, which the --cutoff applies to, represents the level of enrichment of the ChIP-seq signal at each genomic position relative to the background. Score 5 means p-value 1e-5 which is calculated during the initial peak calling process. Scores between 2 and 5 are frequently used. A cutoff of 2 is a relatively lenient threshold and a cutoff of 5 is more stringent, thus reducing the number of peaks but increasing their reliability. Differential HP1 enrichment was detected by DiffBind.^21^ IGV was used for visual inspection.

The same procedures were applied to *D. melanogaster* data based on dmel_r6.06_FB2015_03_gtf_dmel-all-r6.06.gff3 and the corresponding genome fasta file. Details in Supplementary data (Table I).

For analysis of *B. glabrata*, the by-catch of the sporocyst ChIPmentation experiments was used, *i.e.*, *Biomphalaria* chromatin that had been immunoprecipitated together with the sporocyst preparation and that represented roughly 25% of the total reads. Alignment was done against all contigs ≥ 5 kb of VectorBase *Biomphalaria-glabrata*-BB02_SCAFFOLDS_BglaB1.fa, and gene annotations were used from *Biomphalaria-glabrata*-BB02_BASEFEATURES_BglaB1.6.gff3. The list of genes was generated with the script, provided in the Supplementary data.

*In vivo studies* - *In vivo* study was carried out with 15 female Balb/C mice divided into three groups. Ethical statement at the Universidade Estadual de Campinas was under number CEUA protocol (Comissão de Ética no Uso de Animais # 6064-1/2022). Each group contained five animals, and each animal was infected with 100 3-day-old schistosomula (Groups 1-3). The quantity of schistosomula and cercariae per animal was determined according to the study by Vilar and collaborators.^22^ The infection of the groups was subcutaneous, however, in groups 1 and 2, represented by SmHP1 and dsmCherry, respectively, the schistosomula were previously incubated in culture for three days with dsRNA. The third group was also kept in culture for three days, however, in the absence of dsRNA. For each group, 100 schistosomula were inoculated per animal. Schistosomula was conducted as previously described.^23^ Briefly, Belo Horizonte (BH) lineage of the *S. mansoni* was used and schistosomula were transformed by tail break and several RPMI washes for tail removal. After three washes, 200 schistosomula were counted and distributed equally in culture plates with 2 mL of Medium 169 (Atená, Biotechnology, Campinas, Brazil), described by,^24^ supplemented with hormones and foetal bovine serum, 30 μg of dsRNA and kept in CO2 atmosphere at 37ºC for three days.

All dsRNAs were done using the T7RiboMAX Express RNAi System (Promega, Belo Horizonte, Brazil). Specifically, the oligonucleotides for amplification HP1 gene (T7 + Forward TAATACGACTCACTATAGGGTGTGGAAGAGTCAGCTGGT and T7 + reverse TAATACGACTCACTATAGGGAGGGTCGATTTTCAGGTGTG), containing T7 promotor for dsRNA synthesis. Gene expressions were evaluated in the QuantStudio qPCR System (Thermo), using the HP1 primers [Forward primer (CGTCACTCAGTTCAGACAGC) and reverse primer (CTCTTCCACACTCACGGGTA)] and endogenous SmEIF4E (Smp_001500) as described previously,^25^ and using the three days-schistosomula condition as gene expression calibrator.^26^

Infected animals with wild type and dsRNA were weighted and Kato Katz^27^ were done one week before perfusion. Perfusion was done as described.^28^ For histology, liver tissues were fixed in 10% formaldehyde and fixed in paraffin blocks and tissue slices were cut and coloured by Masson’s trichrome. All granulomas present in 10 random fields of the histological section of each animal were quantified. The images were captured using a photomicroscope using Leica^®^ LAS EZ4 HD software. The total area of the granuloma was measured using ImageJ 1.53t software.

## RESULTS

*AntiHP1 antibody ab109028 can be used for ChIP in S. mansoni* - Antibody quality is an important criterion for the success and reliability of ChIP-Seq. HP1 is a conserved protein and we have shown before that antiHP1 Abcam ab109028 can be used in Western Blots with *S. mansoni*. A literature search for the use of ab109028 resulted in 31 publications [Supplementary data (Table II)] but only in one case was it used for ChIP-seq, and the efficiency of the antibody was not tested there. Thus, initial experiments were necessary to evaluate if the antibody was suitable for ChIP-Seq. We used a previously developed pipeline to test whether ab109028 (lot numbers GR38873387336-6 and -9) can be used for ChIP-Seq.^29^ Essentially, the method consists of (i) performing a Western blot to assure that only the band of the expected size can be observed and (ii) doing a chromatin IP titration experiment with a constant amount of input chromatin and increasing amounts of antibody to test if saturation of available epitopes can be achieved.

**Fig. 1:**
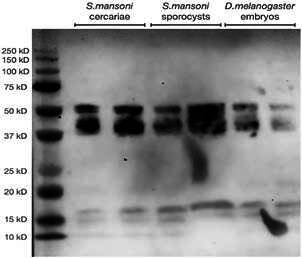
western blot on crude protein extracts of *Schistosoma mansoni* cercariae and sporocysts (left and middle), and *Drosophila melanogaster* embryos (right). All lanes in duplicates. Left lane molecular weight marker.

We performed Western Blots on *S. mansoni* cercariae and sporocysts. We used *D. melanogaster* embryos as positive control because in this model organism HP1 was successfully characterised by Western Blot and ChIP-Seq experiments. Expected molecular weight of *D. melanogaster* HP1 is 23.194 kD.^30^ The dimer is therefore predicted to have a molecular weight of 46 kD.^16^ Such a band is observed in all samples in the Western blot (Fig. 1) but supplementary bands can be distinguished. Apparently, under our relatively gentle extraction conditions and without beta-mercaptoethanol the dimers remained intact. We noticed that this behaviour is not without precedent^31^ ^,^ ^32^ even for Knock-out validated antibodies.^33^ The results shown in Fig. 1 agree also with previous western blotting^16^ which demonstrated that SmHP1 can form dimers in solution, showing a band of approximately 56 kD.

**Fig. 2:**
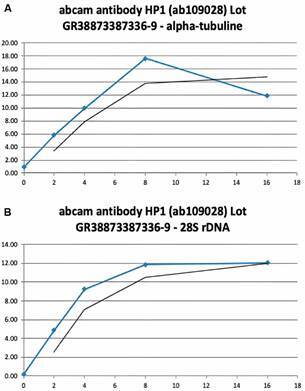
results of titration of antiHP1 antibody. X-axis indicates amount of antibody in µL, Y-axis input recovery in %. Upper panel for Sm-alpha-tubuline, lower panel for Sm-28S-rDNA. Saturation is achieved with 8 µL of antibody. Blue line represents experimental data, black line is the calculated trend line.

We then proceeded to ChIPmentation titration of the antibody using chromatin from *S. mansoni* cercariae. ChIPmentation is a streamlined method of crosslink Chromatin Immunoprecipitation (ChIP) that uses Tn5 for integration of adaptor for library amplification (Tagmentation). A constant amount of chromatin corresponding to 160,000 cells was incubated with 0, 2, 4, 8 and 16 µL of ab109028 during the ChIPmentation procedure and input recovery was measured using qPCR on 2 arbitrarily chosen loci: Sm- alpha-tubuline (Fig. 2A) and Sm-28S-rDNA (Fig. 2B). Results are shown in Fig. 2, indicating that above 8 µL antibody saturation is achieved.

Based on this initial testing we decided to proceed to ChIPmentation with 8 µL of antibody.

**Fig. 3:**
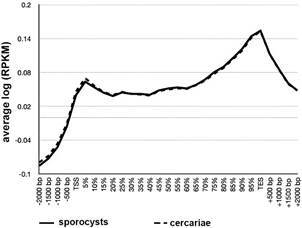
metagene profiles of *Schistosoma mansoni* sporocysts (solid line) and cercariae (dotted line). X-axis: relative position around genes; Y-axis: average log(reads per kilobase per million mapped reads - RPKM) of two replicates; TSS: transcription start site; TES: transcription end site.

*ChIP-Seq metagene profiles in S. mansoni peak around transcription end sites (TES)* - After having firmly established that ab109028 was suitable for ChIP we proceeded to ChIPmentation on cercariae and sporocysts of *S. mansoni* [Supplementary data (Table I)]. We hypothesised that HP1 could establish a heterochromatic structure in the cercariae that are transcriptionally inactive. To investigate the distribution of HP1 with relation to known genomic features we produced metagene profiles around protein coding genes. In contrast to what was observed in other species, SmHP1 shows enrichment around the TES (Fig. 3) in both cercariae and sporocysts.

This unexpected result and the fact that the current study is the first analysis of HP1 distribution and there are therefore no precedents to compare with, prompted us to test the ChIPmentation procedure with antiHP1 ab109028 on the well characterised model species *D. melanogaster*.

*AntiHP1 antibody ab109028 delivers canonical ChIP-Seq results in D. melanogaster* - It might be argued that the metagene profiles observed in *S. mansoni* are due to a peculiar nature of the antibody that remained unnoticed or an experimental error in our ChIPmentation procedure. If this would hold true, then our experiments would deliver results that are different from previously published data. To test this hypothesis we performed ChIPmentation experiments with *D. melanogaster* embryos, a species for which data of ChIP-Seq experiments are available. To obtain these data we used the query terms “hp1 chip *Drosophila*” to search the NCBI SRA and obtained 186 results belonging to 55 BioSamples. We arbitrarily choose ChIP-Seq data for WT embryo replicate 1 and 2 (NCBI SRA: SRS6795886, SRS6795887) corresponding to ENA SRX8497063 and SRX8497065 and data for input (NCBI SRA SRS6795884, ENA SRX8497062). The NCBI SRA entry states that 2 µg antiHP1, Developmental Studies Hybridoma Bank C1A9 had been used.^34^

We also downloaded ChIP-Seq data for fly heads that had been produced with the same antibody^35^ (NCBI BioProject PRJNA490276): ENA SRR7817540, SRR7817541, SRR7817542 for three ChIP-Seq replicates and ENA SRR7817573 for the input.

**Fig. 4:**
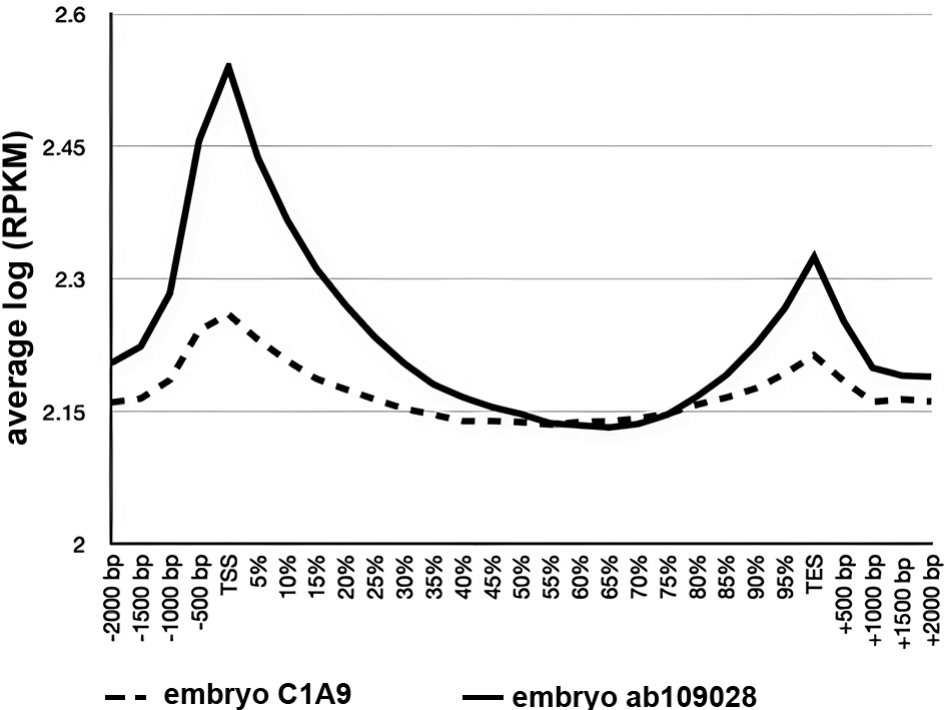
metagene profiles of *Drosophila melanogaster* embryos (solid line) produced in this study with antibody ab109028, and previously published data on embryos (dotted line) with antibody C1A9. X-axis: relative position around genes; Y-axis: average log(RPKM) of two replicates (the values can therefore not be compared directly to y-axis of Figs 3-4).

**Fig. 5:**
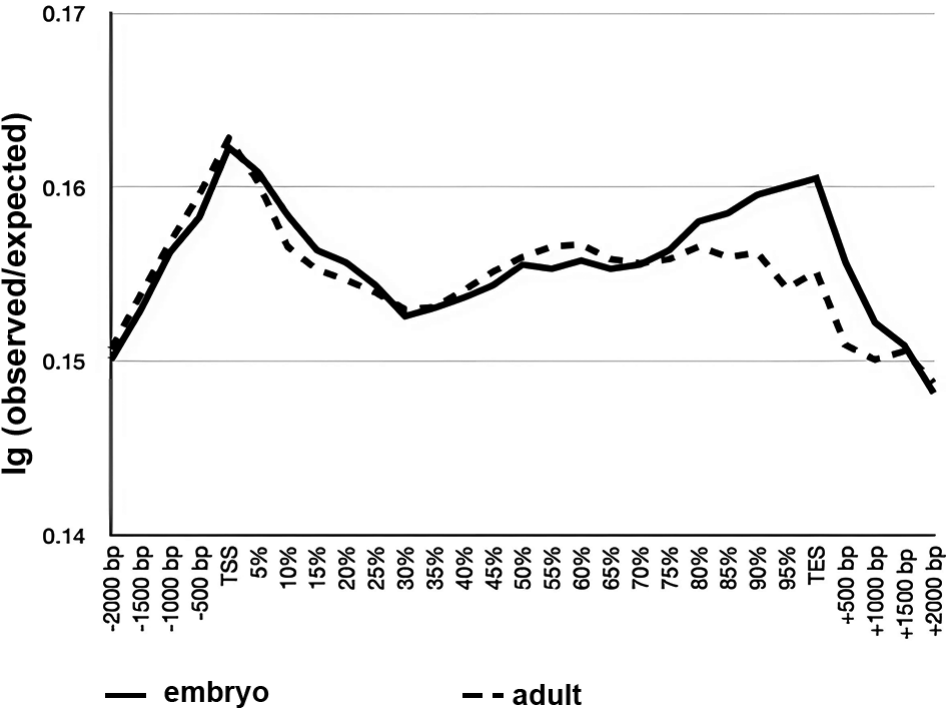
metagene profiles of *Drosophila melanogaster* embryos (solid line) produced in this study with antibody ab109028, and previously published data from another laboratory on adult flies (dotted line) with antibody C1A9. X-axis: relative position around genes; Y- axis log(observed/expected) of two replicates.

We then performed ChIPmentation with antibody ab109028 on fruit fly embryos under the same conditions as for our *S. mansoni* samples. After sequencing, we processed the published data and our experimental data as described for the *S. mansoni* samples. For SRA data of embryos, ChromstaR did not manage to construct a differential model probably due to relatively low enrichment of the reads. We resorted therefore to read counts distributions around metagenes [log(RPKM) instead of log(obs/exp)], shown in Fig. 3. While it is interesting to note that there is a small decrease in adult flies compared to embryos at the TES (Fig. 4), we did not observe strong differences between the profiles generated based on the data of the two independent earlier studies and our experiment (Fig. 5).

Therefore, we conclude that antibody ab109028 delivers canonical ChIP-seq profiles around genes with the fruit fly as a model. Consequently, we have no reason to believe that the non-canonical HP1 chromatin landscape around genes in *S. mansoni* is due to the antibody quality or the ChIPmentation procedure.

While the question was not central to our experiment we profited from the by-catch of ChIP-Seq data from *B. glabrata* in the sporocysts ChIPmentation reads to show if this non-canonical binding pattern is schistosome specific or is a general trait of lophotrochozoans, a clade to which *Biomphalaria* and Schistosomes belong, and that is evolutionary distinct from insects. Results are shown in Supplementary data (Fig. 1) and indicate that *B. glabrata* metagene profiles are similar to *D. melanogaster* with enrichments in the TSS and TES of protein coding genes, and different from *S. mansoni*.

*There are few regions with differential enrichment of HP1 between cercariae and sporocysts* - Since we had established that both the antibody and the new ChIPmentation procedure delivered reproducible results in model organisms we proceeded to comparative analyses between *S. mansoni* live cycle stages. We used ChromstaR to identify 154 regions with differences in HP1 enrichment between cercariae and sporocysts [Supplementary data (Table I)]. However, these regions are small and visual inspection of HP1 landscape suggested that HP1 enrichment occurs over broader regions. We therefore applied another software, MACS bdgbroadcall, on combined uniquely aligned ChIP-Seq reads, independently for sporocysts and cercaria. Results are in Table.

**TABLE:** Number of broad peaks in cercariae and sporocyst larvae using different p-value scores from MACS2

	Number of peaks (p-value 1e-2)	Number of peaks (p-value 1e-5)
Sporocysts	6406	89
Cercariae	7485	222

**Fig. 6:**
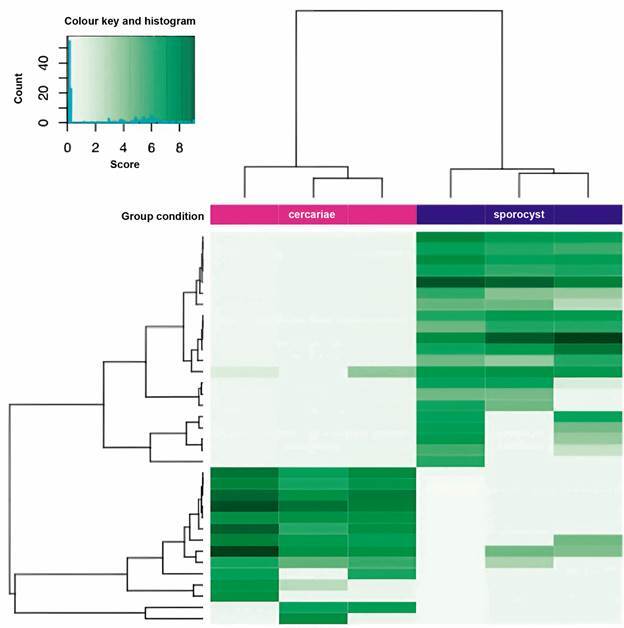
clustering of cercariae (magenta) and sporocyst (purple) larvae based on differential HP1 chromatin enrichment.

We then used DiffBind for the identification of differential enrichment between sporocysts and cercariae. Based on the conservative peakcall at score 5 we identified 36 differentially enriched regions that separated sporocysts and cercariae into 2 clusters (Fig. 6).

Since this number is relatively low, we wondered whether the number of identified differences could have been obtained by chance alone. We used bootstrapping through random segments that matched the 222 broad cercariae HP1 peaks in number and total span and used DiffBind for identification of differential enrichment. Bootstrapping through these analyses found in average 61.5 ± 6.8 differential peaks. Consequently, we conclude that the differences identified by DiffBind identified on broad regions lack the statistical power to be confident about their occurrence other than by accident.

In summary, our findings cannot formally exclude that there are differences between sporocyst and cercariae in HP1 enrichment, but differences are small. To establish a profile of genes which are enriched and regulated by SmHP1 we generated a script (Supplementary data) to generate a gene list which was enriched by SmHP1 protein. The list gene for cercariae [Supplementary data (Table III)] and sporocyst [Supplementary data (Table IV)] showed interesting results. For cercariae, we found genes related to kinases proteins, which suggest the importance of phosphorylation and signalling pathways in cercariae. For sporocyst we also found kinases and other proteins with diverse functions.

**Fig. 7:**
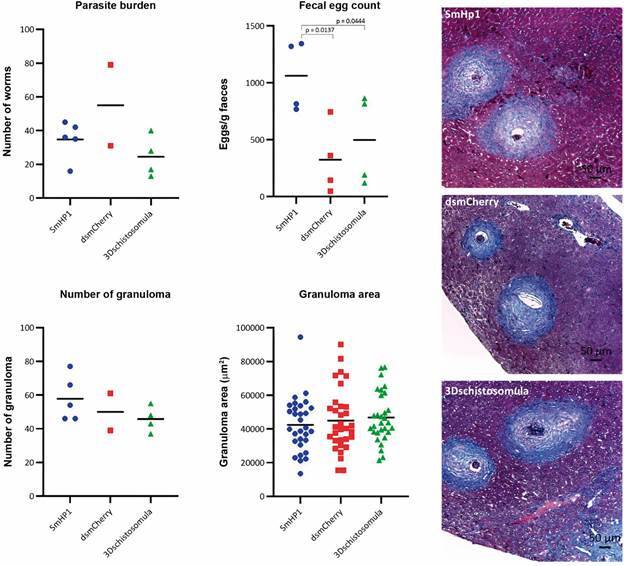
functional in vivo studies in mice injected with (i) dsRNA incubated HP1 three days schistosomula (blue) compared to (ii) mock treated *dsRNA* gene (dsmCherry), and (iii) wild type. Parasite burden, faecal egg count, granuloma number and area were measured. Significant differences were only found for faecal egg count which increased for the knock-down parasites.

*In vivo knock-down of SmHP1 increases parasite’s oviposition in mice* - Given the unexpected distribution of *Sm*HP1 over the genome and absence of important differences between two developmental stages we wondered if *Sm*HP1 played any role in the parasite in vivo. We designed an experiment to perform the knockdown of *Sm*HP1 by RNA interference in schistosomula. Decrease of expression was small [Supplementary data (Fig. 2)] compared with control dsmCherry and wild type schistosomula. *In vivo* results are shown in Fig. 7. Parasite burden showed that knock-down parasites were able to migrate, develop and to achieve the correct destination in portal hepatic and mesenterial veins, despite dsRNA employed and schistosomula injection. To evaluate infection success and parasite fitness, we measured granuloma and egg count/faeces gram using Kato-Katz method.^27^ The number of granulomas and areas were the same for all tested conditions, suggesting that knock-down of *Sm*HP1 does not affect the granulomatous response from the host. Surprisingly, the parasite number of eggs/faeces gram were two times higher in the knock-down and this difference was statistically significant.

## DISCUSSION

Recent years have seen an important increase in our understanding of the chromatin structure of *S. mansoni*. However, this knowledge stems exclusively from work on histone modifications and DNA methylation. To widen our view of other chromatin proteins we focused here on the HP1 homologue.

Our results of antibody titration show you that antibody ab109028 is suitable for ChIP- seq experiments on *S. mansoni*. Our report shows for the first time the HP1 profile for this species. There are several works that have focused on transcription and translation in cercariae, absence of transcription but use of mRNA from previous stages, low rates of translational until infection and schistosomula migration when transcription is activated.^36^ ^,^ ^37^ ^,^ ^38^ Since cercariae is transcriptionally silent and poised for transcription,^4^ we reasoned that they might have a tighter heterochromatin formation and differential HP1 occupancy, but our results suggest the opposite: HP1 presence (and presumably heterochromatinisation) might not be advantageous if it is necessary to rapidly activate transcription after infection.

HP1 is a conserved member of the large family of chromatin proteins, and it is believed to fulfil conserved functions in different organisms. For example, in *Drosophila* there are five isoforms of HP1 and it is mainly located in heterochromatic regions rich in H3K9me3^34^ and preferentially associated with polytene chromosomes and the X chromosome. The study also suggested that the formation of heterochromatin occurs in parallel with the transcriptional activation of the nuclei and expression of different genes.^39^ Previous work using RNA interference technology found that HP1 silencing was related to chromosomal defects.^39^ ^,^ ^40^ The distribution of HP1 throughout the *S. mansoni* genome does not follow a pattern observed for other organisms suggesting that its function is also different.

The choice of *Drosophila* as a comparative model is justified by the fact that there are no previous ChIP-seq studies for SmHP1 in *S. mansoni*. Although *Drosophila* is an organism evolutionarily distant from *S. mansoni*, the functions of this protein are conserved among organisms with link to the trimethylation of lysine 9 in histone H3 (H3K9me3) to maintain the silenced state of chromatin. This binding has been shown to be important for HP1 to perform its functions and studies have shown this binding of HP1 to the epigenetic mark H3K9me3 in yeast,^41^ ^,^ ^42^ *Drosophila*,^43^ ^,^ ^44^ ^,^ ^45^ mammals^46^ ^,^ ^11^ and *Plasmodium*.^10^ ^,^ ^13^ ^,^ ^47^

The conservation of HP1 domains among organisms is a strong indication of conservation of function and, although *Sm*HP1 is conserved and has similarities with other HP1 proteins, it is noteworthy that in *S. mansoni* this protein exerts distinct functions. In *S. mansoni*, *Sm*HP1 can be found in almost the entire genome, spanning also some genes differentially expressed between the studied lifecycle stages. In addition to HP1 binding to H3K9me3 in many organisms, this protein also binds to the epigenetic mark H3K27me3 to produce repressive effects on gene transcription. The study of *Sm*HP1 was therefore based on the hypothesis that *Sm*HP1 is involved in transcriptional silencing in cercariae. Given the evolutionary conservation of the protein’s function, we hypothesised that *Sm*HP1 operates through the same mechanism of action that has been observed in other organisms. Immunoprecipitation and mass spectrometry data from a previous study^16^ showed that *Sm*HP1 immunoprecipitated with the PRMT1 protein, an arginine methyltransferase. This protein is highly homologous to the vertebrate PRMT1 enzyme and is responsible for methylating histone H4 and plays a role in nuclear receptor- mediated chromatin remodelling.^48^ These results, together with the fact that no methyltransferases responsible for H3K9 methylation were co-immunoprecipitated with SmHP1, corroborate the narrative that SmHP1 plays distinct functions in *S. mansoni* based on the methylation of histones other than H3.

In addition, we performed a bioinformatics analysis that delivered a gene list that matched genes with the enrichment of *Sm*HP1 in cercariae and schistosomula. The list was enriched for genes coding for protein kinases and which are therefore possibly regulated by HP1 in cercariae and sporocyst. Recent study reviewed protein kinases functions in Schistosomes.^49^ These proteins were engaged in homeostasis processes in the parasite. Moreover, transcriptome analysis was reviewed and uncovered several genes which were expressed in cercariae and in the whole life cycle.^49^ Taken together, our results and the transcriptome analyses may reinforce the notion that kinases are important for cercariae homeostasis and life cycle progression of *S. mansoni*.

Our *in vivo* experiments showed that even with a moderate decrease in *Sm*HP1 expression it was possible to observe an increase in oviposition. This goes in line with earlier results where alteration of oviposition in adult worms’ *in vitro* culture with siRNA against *Sm*CBX/*Sm*HP1 (Smp_179650). In that work, it was also demonstrated that *Sm*CBX/*Sm*HP1 interacts with putative methylated DNA binding protein *Sm*mbd2/3 colocalising together in Schistosome neoblasts and reproductive tissues, suggesting a role in the reproductive organs of parasite.^17^

Our results represent another step towards a better understanding of the role of *Sm*HP1 in chromatin structure, gene expression and parasite fitness. It shows that results from model organisms, albeit tremendously useful in many cases, cannot be simply extrapolated to any other organism. *Sm*HP1 seems to play according to rules that are yet to be discovered.
