# Supplementary material for: Heterochromatin protein 1 (HP1) of Schistosoma mansoni: non-canonical chromatin landscape and oviposition effects
Source: Mem Inst Oswaldo Cruz. 2025 Mar 31;120:e240075. doi: 10.1590/0074-02760240075 (PMC11961034; doi:10.1590/0074-02760240075)
Supplement: Supplementary file 1 [file 1678-8060-mioc-120-e240075-s1.pdf]

```
#!/bin/bash

# Step 1: Filter genes longer than 200 nucleotides and extract relevant columns
#awk '{if ($3-$2>200){print $0}}' MACS2_bdgbroadcall_on_combined_Cer-D-U_cut-off_5.bed | cut -f1,2,3 | sort -k1,1 -k2,2n > genes_sorted_fran.bed
awk '{if ($3-$2>200){print $0}}' MACS2_bdgbroadcall_on_combined_Sp-D-U_cut-off_5.bed | cut -f1,2,3 | sort -k1,1 -k2,2n > genes_sorted_fran.bed
echo "Filtered peaks longer than 200 nucleotides and saved to genes_sorted_fran.bed"

# Step 2: Select gene-related lines from the annotation file and extract relevant columns
awk '{if ($3 == "gene"){print $0}}' schistosoma_mansoni.PRJEA36577.WBPS17.annotations.gff3 | cut -f1,4,5,9 | sort -k1,1 -k2,2n > genes_all.bed
echo "Selected gene-related lines and saved to genes_all.bed"
echo "Linhas relacionadas aos genes selecionadas e salvas em genes_all.bed"

# Step 3: Perform intersection to obtain regions that overlap between both files
multiIntersectBed -i genes_sorted_fran.bed genes_all.bed -header -names A B | awk '{if ($5 == "A,B"){print $0}}' | cut -f1,2,3 > resultnew.bed
echo "Performed intersection and saved the overlapping regions to resultnew.bed"
echo "Interseção realizada e regiões sobrepostas salvas em resultnew.bed"

# Step 4: Add gene annotation related to each region
bedtools intersect -a genes_all.bed -b resultnew.bed -wb | cut -f1,2,3,4 > FINAL.bed
echo "Added gene annotation and saved to FINAL.bed"
echo "Anotação gênica adicionada e salva em FINAL.bed"
```

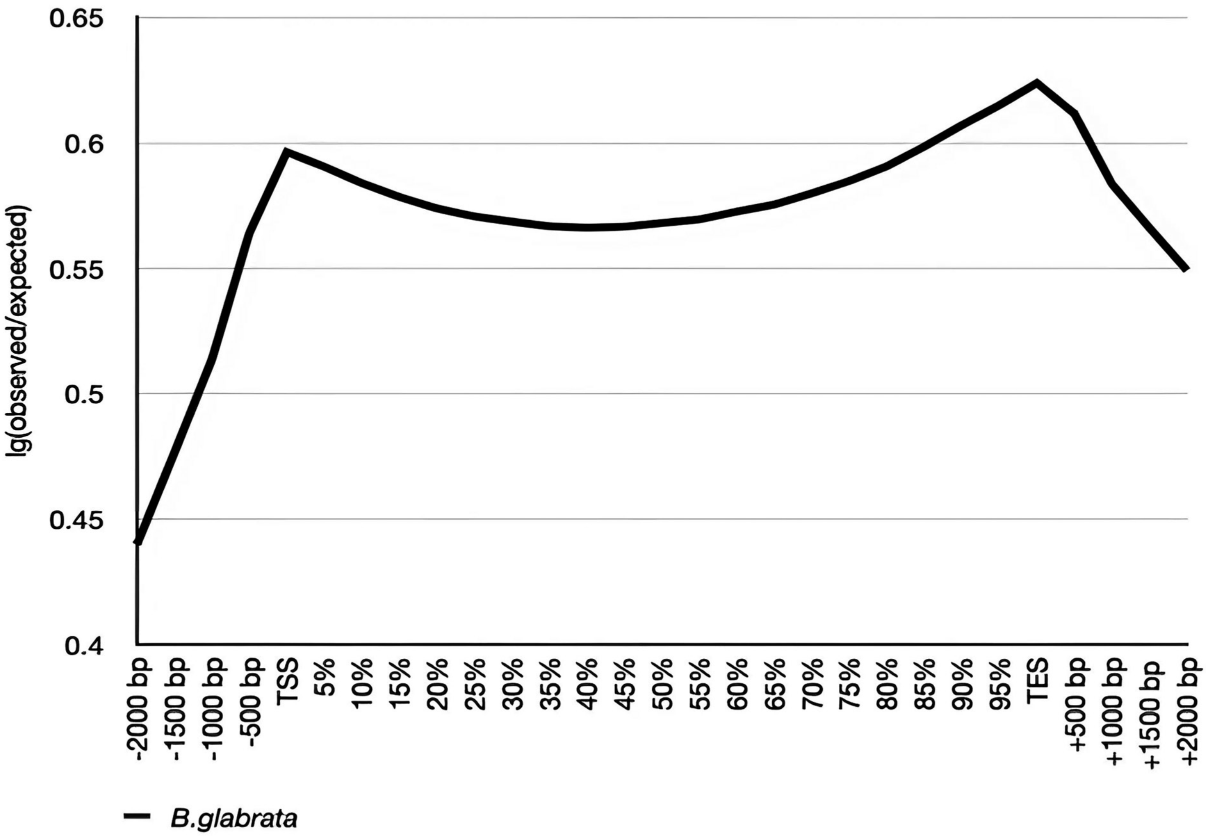

Fig. 1: metagene profiles of *Biomphalaria glabrata* adults. X-axis: relative position around genes; Y-axis log(observed/expected) of three replicates.

cc BY

TABLE I

Detailed sequencing and alignment data

Sample	Supplier name	File name	readsR1	Adapter trim ming	Aligne - mentrate	After pick unique and RmDup	In %	Down - sample to
Ce 1	cercaria ChIP HP1 - replica 1	Ce1_S25_L001_R1_001_fastq_gz	27,584,702	100%	85.75%	8,539,009	30.96%	4,700,000
Ce 2	cercaria ChIP HP1 - replica 2	Ce2_S26_L001_R1_001_fastq_gz	31,243,784	100%	90.78%	10,362,799	33.17%	4,700,000
Ce 3	cercaria ChIP HP1 - replica 3	Ce3_S27_L001_R1_001_fastq_gz	30,792,782	100%	81.45%	8,857,714	28.77%	4,700,000
Ce-In	input cercariaeundiluted Tn5	InNa_S31_L001_R1_001_fastq_gz	14,441,656	100%	96.04%	9,316,089	64.51%	4,700,000
Sp 1	sporocyst ChIPHP1 - replica 1	Sp1_S28_L001_R1_001_fastq_gz	34,896,078	100%	59.46%	7,340,487	21.04%	4,700,000
Sp 2	sporocyst ChIPHP1 - replica 2	Sp2_S29_L001_R1_001_fastq_gz	35,630,630	100%	61.87%	7,967,706	22.36%	4,700,000
Sp 3	sporocyst ChIPHP1 - replica 3	Sp3_S30_L001_R1_001_fastq_gz	29,439,162	100%	44.01%	4,719,547	16.03%	4,700,000
Sp-In	input tagmentationlibrarysporocysts SmDFO inBgGUA	Sp- In_S7_L001_R1_001_fastq_gz	188,264,064	100%	57.44%	16,001,570	8.50%	4,700,000
Dm2	*D. melanogaster* embryos HP1 ChIPmentation rep 2	Dm2_S9_L001_R1_001_fastq_gz	157,680,688	100%	83.54%	18,515,265	11.74%	16,000,000
Dm3	*D. melanogaster* embryos HP1 ChIPmentation rep 3	Dm3_S10_L001_R1_001_fastq_gz	185,909,252	100%	87.05%	22,123,566	11.90%	16,000,000
Dm-In	*D. melanogaster* embryos HP1 Input	Dm-In_S11_L001_R1_001_fastq_gz	173,301,784	100%	95.84%	20,385,745	11.76%	16,000,000
Dm-adult1	*D. melanogaster* adults HP1 ChIP rep 1 from SRA	SRR7817540.fastq.gz	27,849,221	100%	91.26%	12,676,124	45.52%	10,000,000
Dm-adult2	*D. melanogaster* adults HP1 ChIP rep 2 from SRA	SRR7817541.fastq.gz	30,240,617	100%	91.42%	15,525,830	51.34%	10,000,000
Dm-adult3	*D. melanogaster* adults HP1 ChIP rep 3 from SRA	SRR7817542.fastq.gz	37,549,684	100%	89.03%	10,173,605	27.09%	10,000,000
Dm-input	*D. melanogaster* adults HP1 Input from SRA	SRR7817573.fastq.gz	23,583,211	100%	95.44%	17,566,290	74.49%	10,000,000
Dm-embryo1	*D. melanogaster* embryo HP1 ChIP rep 1 from SRA	SRR11952662.fastq.gz	19,894,682	100%	94.63%	12,376,186	62.21%	10,000,000
Dm-embryo2	*D. melanogaster* embryo HP1 ChIP rep 2 from SRA	SRR11952664.fastq.gz	46,050,252	100%	91.61%	25,623,510	55.64%	10,000,000
Dm- embryo-In	*D. melanogaster* embryo HP1 input from SRA	SRR11952661.fastq.gz	27,346,764	100%	95.32%	19,767,614	72.29%	10,000,000
Sp 1	sporocyst ChIPHP1 - replica 1	Sp1_S28_L001_R1_001_fastq_ gz	34,896,078	100%	24.34% on*B. glabrata*	5,387,400	15.44%	4,600,000
Sp 2	sporocyst ChIPHP1 - replica 2	Sp2_S29_L001_R1_001_fastq_gz	35,630,630	100%	20.29% on *B. glabrata*	4,600,990	12.91%	4,600,000
Sp 3	sporocyst ChIPHP1 - replica 3	Sp3_S30_L001_R1_001_fastq_ gz	29,439,162	100%	31.97% *on B. glabrata*	6,069,096	20.62%	4,600,000
Sp-In	input tagmentationlibrary sporocysts SmDFO in BgGUA	Sp- In_S7_L001_R1_001_fastq_gz	188,264,064	100%	31.93% on*B. glabrata*	39,540,484	21.00%	4,600,000

TABLE II
Previous use of antiHP1 antibody Abcam ab109028

Designation of target	Reference	Technique	Cat# (Abcam)
HP1α	(50)	Cell Tension	ab109028
CBX5	(51)	Chemiprecipitation	ab109028
HP1α	(52)	ChIP-seq	ab109028
HP1α	(53)	Co-Immunoprecipitation	ab109028
HP1α	(54)	eCLIP-seq	ab109028
HP1α	(55)	Immunoblots	ab109028
HP1α	(56)	Immunoblotting and immunostaining	ab109028
HP1α	(57)	immunoblotting, immunofluorescence, flow cytometry	ab109028
HP1α	(58)	Immunocytochemistry	ab109028
HP1α	(59)	Immunofluorescence	ab109028
HP1	(60)	Immunofluorescence	ab109028
HP1α	(61)	Immunofluorescence	ab109028
HP1α	(62)	Immunofluorescence	ab109028
HP1α	(63)	Immunofluorescence	ab109028
HP1α	(64)	Immunofluorescence	ab109028
HP1α	(65)	Immunofluorescence	ab109028
HP1α	(66)	Immunofluorescence	ab109028
HP1α	(67)	Immunofluorescence	ab109028
HP1α	(68)	Immunofluorescence	ab109028
HP1α	(69)	Immunofluorescence	ab109028
HP1α	(70)	Immunofluorescence	ab109028
HP1α	(71)	Immunofluorescence and Western Blotting	ab109028
HP1α	(72)	Immunofluorescence and Western Blotting	ab109028
HP1α	(73)	Immunohistochemistry	ab109028
HP1α	(74)	Immunoprecipitation and Western blotting	ab109028
CBX5	(75)	Western Blotting	ab109028
HP1	(76)	Western Blotting	ab109028
HP1	(77)	Western Blotting	ab109028
CBX5	(78)	Western Blotting	ab109028
HP1	(79)	Western Blotting	ab109028
HP1α	(80)	Western Blotting and immunoprecipitation	ab109028

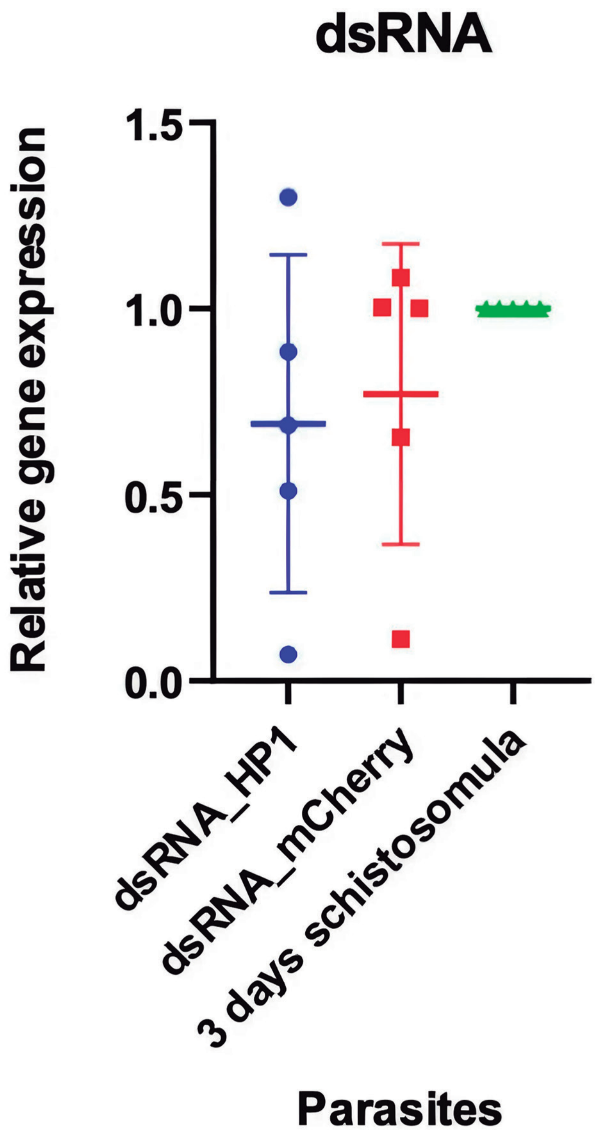

Fig. 2: relative gene expression of dsRNA schistosomula culturescompared to expression of wild type schistosomula. The gene expression was calculatedby delta CT method,[26] using wild type schistosomula as gene expression calibrator andthe endogenous gene was SmEIF4E (Smp_001500) as described previously.[25]

REFERENCES

50. Deshpande P, Prentice E, Ceballos AV, Casaccia P, Elbaum-Garfinkle S. Modified histone peptides uniquely tune the material properties of HP1α condensates. bioRxiv [Preprint]. 2024. Available from: https://pubmed.ncbi.nlm.nih.gov/38370661/.

51. Lamb KN, Dishman SN, Waybright JM, Engelberg IA, Rectenwald JM, Norris- Drouin JL, et al. Discovery of potent peptidomimetic antagonists for heterochromatin protein 1 family proteins. ACS Omega. 2021; 7(1): 716-32.

52. Zhao H, Lin Y, Lin E, Liu F, Shu L, Jing D, et al. Genome folding principles revealed in condensin-depleted mitotic chromosomes. bioRxiv [Preprint]. 2023. Available from: https://pubmed.ncbi.nlm.nih.gov/38014261/.

53. Wistner SC, MacDonald IA, Stanley KA, Hathaway NA. Characterization of hepatoma-derived growth factor-related protein 2 interactions with heterochromatin. Cells. 2023; 12(2): 325.

54. Zhang X, Jiang Q, Li J, Zhang S, Cao Y, Xia X, et al. KCNQ1OT1 promotes genome-wide transposon repression by guiding RNA-DNA triplexes and HP1 binding. Nat Cell Biol. 2022; 24(11): 1617-29.

55. Gomes AP, Ilter D, Low V, Rosenzweig A, Shen ZJ, Schild T, et al. Dynamic incorporation of histone H3 variants into chromatin is essential for acquisition of aggressive traits and metastatic colonization. Cancer Cell. 2019; 36(4): 402-417.e13.

56. Strom AR, Biggs RJ, Banigan EJ, Wang X, Chiu K, Herman C, et al. HP1α is a chromatin crosslinker that controls nuclear and mitotic chromosome mechanics. Elife. 2021; 10: e63972.

57. Heath J, Cheyou ES, Findlay S, Luo VM, Carpio EP, Lee J, et al. POGZ promotes homology-directed DNA repair in an HP1-dependent manner. EMBO Rep. 2022; 23(1): e51041.

58. Cappuyns E, Huyghebaert J, Vandeweyer G, Kooy RF. Mutations in ADNP affect expression and subcellular localization of the protein. Cell Cycle. 2018; 17(9): 1068-75.

59. Ren X, Tong Y, Yang T, Huang S, Xu T, Xue Q, et al. Overexpression of BRG1 improves early development of porcine somatic cell nuclear transfer embryos. Theriogenology. 2024; 217: 51-63.

60. Lionetti MC, Bonfanti S, Fumagalli MR, Budrikis Z, Font-Clos F, Costantini G, et al. Chromatin and cytoskeletal tethering determine nuclear morphology in progerin- expressing cells. Biophys J. 2020; 118(9): 2319-32.

61. Kim T, Yoo J, Do S, Hwang DS, Park Y, Shin Y. RNA-mediated demixing transition of low-density condensates. Nat Commun. 2023; 14(1): 2425.

62. Liu X, Jiang S, Ma L, Qu J, Zhao L, Zhu X, et al. Time-dependent effect of 1,6- hexanediol on biomolecular condensates and 3D chromatin organization. Genome Biol. 2021; 22(1): 230.

63. Jiang H, Bian W, Sui Y, Li H, Zhao H, Wang W, et al. FBXO42 facilitates notch signaling activation and global chromatin relaxation by promoting K63-linked polyubiquitination of RBPJ. Sci Adv. 2022; 8(38): eabq4831.

64. van Bueren MAE, Janssen A. The impact of chromatin on double-strand break repair: imaging tools and discoveries. DNA Repair (Amst). 2024; 133: 103592.

65. Chu Y, Jiang Z, Gong Z, Ji X, Zhu M, Shang Q, et al. PML-mediated nuclear loosening permits immunomodulation of mesenchymal stem/stromal cells under inflammatory conditions. Cell Prolif. 2024; 57(4): e13566.

66. Damodaran K, Venkatachalapathy S, Alisafaei F, Radhakrishnan AV, Sharma JD, Shenoy VB, et al. Compressive force induces reversible chromatin condensation and cell geometry-dependent transcriptional response. Mol Biol Cell. 2018; 29(25): 3039-51.

67. Kong X, Chen G, Li C, Wu X. Molecular characterization of baculovirus-induced chromatin marginalization and architectural alteration. bioRxiv [Preprint]. 2023. Available from: https://doi.org/10.1101/2023.07.17.549271.

68. Carnie CJ, Armstrong L, Sebesta M, Ariza A, Wang X, Graham E, et al. ERCC6L2 mitigates replication stress and promotes centromere stability. Cell Rep. 2023; 42(4): 112329-9.

69. Lyons H, Veettil RT, Pradhan P, Fornero C, De N, Ito K, et al. Functional partitioning of transcriptional regulators by patterned charge blocks. Cell. 2023; 186(2): 327-45.

70. Baird MA, Fischer RS, Jewett CE, Malide D, Cartagena-Rivera AX, Waterman CM. Lamin B receptor upregulation in metastatic melanoma causes cholesterol-mediated nuclear envelope fragility. bioRxiv [Preprint]. 2023. Available from: https://doi.org/10.1101/2023.12.21.572889.

71. Xie X, Almuzzaini B, Drou N, Kremb S, Yousif A, Farrants AÖ, et al. β-Actin- dependent global chromatin organization and gene expression programs control cellular identity. FASEB J. 2018; 32(3): 1296-314.

72. Zaidan NZ, Walker KJ, Brown JE, Schaffer LV, Scalf M, Shortreed MR, et al. Compartmentalization of HP1 proteins in pluripotency acquisition and maintenance. Stem Cell Reports. 2018; 10(2): 627-41.

73. Meseure D, Vacher S, Boudjemaa S, Laé M, Nicolas A, Leclere R, et al. Biopathological significance of PIWI–piRNA pathway deregulation in invasive breast carcinomas. Cancers (Basel). 2020; 12(10): 2833.

74. Nakao M, Sato Y, Aizawa A, Kimura H. Mode of SUV420H2 heterochromatin localization through multiple HP1 binding motifs in the heterochromatic targeting module. Genes to Cells. 2024; 29(5): 361-79.

75. Yan Z, Yang Q, Xue M, Wang S, Hong W, Gao X. YY1-induced lncRNA ZFPM2- AS1 facilitates cell proliferation and invasion in small cell lung cancer via upregulating of TRAF4. Cancer Cell Int. 2020; 20: 108.

76. Yang B, Sun L, Liang L. LncRNA HOXC-AS3 suppresses the formation of mature miR-96 in ovarian cancer cells to promote cell proliferation. Reprod Sci. 2021; 28(8): 2342-9.

77. Ulianov SV, Velichko A, Magnitov MD, Luzhin A, Golov AK, Ovsyannikova N, et al. Suppression of liquid–liquid phase separation by 1,6-hexanediol partially compromises the 3D genome organization in living cells. Nucleic Acids Res. 2021; 49(18): 10524-41.

78. Zhou F, Chen L, Lu P, Cao Y, Deng C, Liu G. An integrative bioinformatics investigation and experimental validation of chromobox family in diffuse large B-cell lymphoma. BMC Cancer. 2023; 23(1): 641.

79. Pandey S, Simmons Jr GE, Malyarchuk S, Calhoun TN, Pruitt K. A novel MeCP2 acetylation site regulates interaction with ATRX and HDAC1. Genes Cancer. 2015; 6(9-10): 408-21.

80. Napoletano F, Bravo GF, Voto IAP, Santin A, Celora L, Campaner E, et al. The prolyl-isomerase PIN1 is essential for nuclear Lamin-B structure and function and protects heterochromatin under mechanical stress. Cell Rep. 2021; 36(11): 109694.
